# Knowledge, Treatment, Control, and Risk Factors for Hypertension among Adults in Southern Iran

**DOI:** 10.1155/2015/897070

**Published:** 2015-12-09

**Authors:** Sayed Fazel Zinat Motlagh, Reza Chaman, Sayed Rashid Ghafari, Zafar Parisay, Mohamad Reza Golabi, Ahmad Ali Eslami, Amin Babouei

**Affiliations:** ^1^Social Determinants of Health Research Center, Yasuj University of Medical Sciences, Yasuj, Iran; ^2^Department of Community Medicine, School of Medicine, Yasuj University of Medical Sciences, Yasuj, Iran; ^3^Department of Health Education and Promotion, School of Health, Isfahan University of Medical Sciences, Isfahan, Iran

## Abstract

Hypertension is the first and the most common risk factor to diseases such as cardiovascular, stroke, and renal diseases. The aim of this study was to determine the factors relevant to hypertension knowledge, treatment, and control in southern Iran. In this cross-sectional study, conducted in Kohgiluye Boyer-Ahmad province, south of Iran, a total of 1836 hypertension patients were randomly selected to participate voluntarily in the study. Hypertension treatment and its control were defined during study. In addition, knowledge about hypertension was measured by hypertension knowledge level scale (HK-LS). Treatment rates were 75.5 and 37.7 percent for female and male, respectively. Habitat, education, income, family history with hypertension, smoking, and time of diagnosis to the disease were found to be related to the treatment of the disease. Control rates were 30.7 and 31.4 for males and females, respectively. Habitat, education, and time of diagnosis to the disease were related to control. Over 50 percent of patients had average knowledge on hypertension. Considering the low rate of control and knowledge on hypertension among patients, health care providers should reinforce their services to improve appropriate knowledge level among elders and, also, plan comprehensive programs to promote health in order to encourage patients change and reform their life style.

## 1. Introduction

Rapid rise of noncommunicable diseases has been considered as a major health challenge in the present century, which threatens social and economic development of communities and people health [1]. Such diseases impose half of the burden of diseases cost in the world [2–4]. Following the present trend, it is predicted that patients will be responsible for over 70 percent of diseases by 2020 [1, 4, 5]. In this regard, hypertension has been considered as the first and the most common risk factor to cardiovascular diseases, stroke, and renal diseases; it has been known as a main modulated disability cause around the world [6, 7] so that hypertension caused at least 45 percent of mortality due to cardiovascular problems and 51 percent of stroke mortality rate [8]. Among 17 million deaths due to cardiovascular problems in the world [8], 9.4 million occur as a consequence of complications of hypertension [9]. In this regard, in Eastern Mediterranean Region where Iran is located, 26 percent of mortality results from hypertension [10].

On the other hand, statistics showed an increase in the prevalence of hypertension. The number of patients with hypertension has increased from 600 million cases in 1980 to 1 billion in 2008; over 40 percent of adults were known to have hypertension [11]. Also, it was suggested that, by 2025, 1.54 billion adults will suffer from hypertension [12]. In this regard, it was reported to be 14 to 34 percent in Iran [13–18] and various studies in other countries reported high rates of hypertension from 18 to 72 percent among males and females [19–27].

It is essential to control hypertension to minimize the side effects of hypertension. Rates reported for hypertension control were disappointing [28], which were suggested to be 13 to 56 percent around the world [23, 26, 29, 30]. An important component to control hypertension is knowledge, which is relative to lower rates of ceasing interventions, following the interventions behavior and better control on disease by patients. As a result, careful evaluation of hypertension has been considered as an inseparable part of general care of the patients [31].

Studies suggested low levels of knowledge on hypertension among patients [23, 24, 32], and lack of correct information and improper understanding of hypertension did not appertain to rural sites; it has been widely reported in urban environments and industrial countries, too [33, 34]. In Iran, studies on knowledge, treatment, and control of hypertension suggested low levels of the mentioned factors [15, 16]. In Isfahan healthy heart program, the effects of comprehensive self-care programs on improving knowledge, attitude, and treatment among patients with hypertension were investigated. The results from the program showed that factors such as knowledge, treatment pursuit, and hypertension control were 49.9, 43.8, and 15.8 percent, respectively [16]. Another study reported treatment and hypertension control as 33.35 and 35.10 percent [15].

Considering factors related to knowledge and hypertension management is an essential starting point to preventing high rates of cardiovascular mortality due to hypertension [35]. However, there is little information available on knowledge, treatment, and control of hypertension in southern Iran. Noticing the importance of the topic, the aim of this study was to determine the factors relevant to hypertension knowledge, treatment, and control in southern Iran.

## 2. Methods

### 2.1. Participants

This cross-sectional study was conducted on a sample of men and women with hypertension in urban and rural health centres in Kohgiluyeh and Boyer-Ahmad Province in southern Iran, in 2014. Patients met the inclusion criteria (six months past the definitive diagnosis disease doctor, a record in health centres, over the age of 30). To participate in the study the patients were invited by phone or visiting their location. Participants who could not communicate effectively with the study personnel or provide informed consent were excluded. Finally, out of the 2,400 patients invited to study, 1836 (76.52%) signed the consent form and voluntarily agreed to participate in the study.

### 2.2. Measures

Questionnaire included three sections that comprised the following questions: sociodemographic characteristics, hypertension-related information, and hypertension knowledge.

#### 2.2.1. Sociodemographic Characteristics Variables

Sociodemographic data included data on age, gender, educational level (illiterate, elementary school, middle school, high school, diploma, and university), monthly family income (>5 million rials, 5 million rials to 10 million rials, 10 million rials to 15 million rials, and <15 million rials), marital status (single, married, divorced, or widowed/widower), employment status which was coded in 5 categories (employed, unemployed, farmer, retired, and housekeeper), physical activity, and smoking. (For smoking and hookah, nonsmoker and smoker were distinguished.)

*Body Mass Index*. Height (height was evaluated with an error close to 0.1 cm, using a SECA portable stadiometer with the individual in the standing position) and weight (weight measurements were taken with participants wearing light clothing and no shoes; weight was measured with an error close to 0.5 Kg using a SECA digital scale) were recorded by a nurse. Body mass index (BMI; calculated as weight/height^2^) was divided into 4 categories: below normal weight (BMI < 18.5), normal weight (18.5 ≤ BMI < 25.0), overweight (25.0 ≤ BMI < 30.0), and obese (BMI ≥ 30.0) [36].

#### 2.2.2. Hypertension-Related Information

Hypertension-related questions included duration of hypertension, family history of HTN, and treatment of HTN which was defined as self-reported current use of antihypertensive medication. Control of HTN was defined as hypertensive participants' SBP < 140 mm Hg and DBP < 90 mm Hg among hypertensive participants who treated their HTN with drugs; that is, control in treatment was also analyzed. Hypertension was defined as systolic blood pressure (SBP) C140 mm Hg or diastolic blood pressure (DBP) C90 mm Hg according to the WHO criteria or history of previously diagnosed disease [37]; BP was measured in a sitting position after a minimum of 5 minutes of rest by using a standardized digital BP measuring machine (Omron Digital).

#### 2.2.3. Hypertension Knowledge

Hypertension knowledge was ascertained by using Hypertension Knowledge Level Scale (HK-LS). This tool was developed from the Hypertension Knowledge Level Scale (HK-LS), a 22-item scale prepared by Erkoc et al. [37]. Hypertension Knowledge Level Scale (HK-LS) assessed respondents' knowledge in defining hypertension, lifestyle, medical treatment, drug compliance, diet, and complication of hypertension. Each item was a full sentence that was either correct or incorrect. And each item was prepared as part of a standard answer (correct, incorrect, or do not know). To assess the content validity of the scale to identify whether items were or were not representative of the knowledge level of hypertension, the opinions of experts were requested via an assessment form. The expert opinions were evaluated, and 2 items were excluded from the scale. All items were evaluated in terms of clarity and expression by considering the expert opinions, and relevant changes (definition (1 item), medical treatment (4 items), drug compliance (4 items), lifestyle (4 items), diet (2 items), and complications (5 items)) were made.

### 2.3. Statistical Analysis

Statistical analyses were performed using SPSS windows version 21.0 software (SPSS Inc., Chicago, Illinois, USA). *P* value < 0.05 was considered significant.

## 3. Results

The mean age of respondents was 66.26 years [SD: 12.36], ranging from 30 to 93 years. Almost, 722 participants were male (39.3%) and 1114 were female (60.7%). More than half of the participants (51.5%) were older than 60. Furthermore, 822 participants (44.8%) were living in cities and 1014 (55.2%) were living in villages. Regarding education, 1149 (66.2%) were illiterate, 362 (19.7%) were at elementary levels, 114 (6.2%) were at guidance level, 46 (2.5%) were at high school level, 90 (4.9%) finished high school, and 75 (4.1%) had academic education. With respect to marital status, 1488 participants (81%) were married. 191 (17.6%) were widows and 25 (1.4%) were single. Over 90 percent of participants had low income. In addition, 45.5 percent reported family history of hypertension. Also, 27.5 percent of the respondents reported regular physical activity (Table 1).

### 3.1. Hypertension Treatment and Control

As shown in Table 1, 222 male (30.7%) and 350 (31.4%) participants reported self-control on their hypertension. There was no significant difference between male and female participants (*P* = 0.796), though it was higher among females. There was a statistically significant correlation between habitat (*P* = 0.000), education (*P* = 0.000), income (*P* = 0.002), family history on hypertension (*P* = 0.003), smoking (*P* = 0.006), and diagnosis time (*P* = 0.045). Participants living in cities, with higher education and income levels, showed better control on their hypertension.

Treatment rate was high among participants. The treatment rates were 75.5% and 37.7% for females and males, respectively, while there was no statistically significant correlation between male and female participants (*P* = 0.383). 36.1 percent of participants who were under treatment showed regular use of their medications. 24.4 percent of participants under treatment reported control on their hypertension. There was a statistically significant correlation between age (*P* = 0.000), habitat (*P* = 0.019), education (*P* = 0.004), and diagnosis time (*P* = 0.045).

### 3.2. Knowledge

Table 2 represents patient's knowledge about hypertension. In general, 25.2 percent of participants showed high knowledge on hypertension (definition of hypertension, treatment pursuit, lifestyle, nutrition, and hypertension side effects).

This parameter was higher among male in comparison to females, participants with higher income in comparison to those with low incomes, among employees in comparison to other occupations, and among those with regular physical activities which was statistically meaningful.

## 4. Discussion

Improving knowledge, treatment, and control on hypertension could decrease high rates of mortality by cardiovascular diseases [38]. Results from our study showed that over half of the participants in the study were over 60 years old. Several studies reported a direct significance in the prevalence of hypertension and age, which corresponded to the results from the present study [14, 39–42]. The results, also, suggested higher hypertension risk with elders. Therefore, it seems essential to plan preventive interventions to control hypertension among older people. Results from the present study reported that hypertension remains mostly undiagnosed during the early years of prevalence so that over 45 percent of patients over 60 were diagnosed for hypertension less than 5 years ago, which suggest the careful consideration of screening and increasing public knowledge on hypertension and its symptoms.

Our results indicated treatment and control rates on hypertension to be 74.8 and 31.2 percent, which were higher in comparison to the results by Esteghamati et al. [16] (2007) in Isfahan where treatment and control on hypertension were 43.8 and 15.8 percent, respectively. This increase could result from implementing Family Physician program and offering health care services to the patients in Iran.

Chow et al. (2013) reported that the rate of treatment and control of hypertension in high-, average-, and poor-income countries is low [43]; results from the studies in Asia suggested similar findings [26, 44, 45]. Low rates of control and treatment of hypertension have been considered as a major risk to increase cardiovascular diseases and stroke [46], which suggested the necessity of efficient implementation of proper training programs and offering essential services to health care services by doctors and health experts, while majority of patients with diagnosed hypertension showed poor control on their disease. This gap has also been reported in several other studies [41, 43, 47]. It seemed that the only way used to control hypertension by patients was taking prescribed medicines; they avoided self-care behaviors such as regular physical activities, good nutrition, and weight control.

Results from the present study reported higher treatment and control of hypertension in females in comparison to males, which was in accordance with the results from other studies [14, 43, 47, 48]. As the patients grew older, the treatment of hypertension improved. It seems that patients were more sensitive to their disease when they grew older and tried medicines to treat their disease. It corresponded to the results from other studies [41, 43, 47].

Another finding from the present study suggested a statistically significant correlation between hypertension control and patients education. The higher their education level, the higher the control rate among patients so that patients having university degrees showed higher control on their hypertension which was in accordance with results from other studies [14, 26, 47, 49], though it was different from study by CHPSNE which reported lower control on hypertension among individuals with lower education [50]. More than half of the participants reported average knowledge on hypertension, treatment pursuit, lifestyle, nutrition, and complication of hypertension. Only 25.2 percent of participants showed high knowledge on the disease. In comparison with other studies, the present study reported lower rates on the issue [34, 51–53]. Parmar et al. showed good rates of knowledge on hypertension among participants [52]. The difference could result from high number of illiterate participants living in villages.

Another finding from the present study considered statistically significant correlation between patient's age and knowledge on the disease. It reported that the older the participants the less their knowledge on the issue, which was in accordance with results from other studies [34, 52]. It was concluded that age group of 30 to 39 had the higher knowledge on hypertension in comparison to other age groups, which could result from their higher education levels relative to other groups. Results from the present study in this regard suggested a meaningful relationship between education and knowledge: as the education among the patients improves, their knowledge on hypertension increases. It was in agreement with other studies [34, 52]. Low knowledge levels on hypertension were in relation to old age, low levels of education, and low income. Lack of training materials, poor screening, and poor consistency of health team were reported in these groups. It seems necessary to create proper training materials by health experts to improve patients' knowledge and, also, increase treatment rates and control on patients.

The strength of the present study was investigating a large urban and rural population with high rates of responding. However, as over 80 percent of the participants were over 50 years old and there was not a normal coverage to all possible age groups, it was considered as a limitation to the study.

## 5. Conclusion

Considering the low rate of knowledge and control of patients on hypertension, health care providers should reinforce their activities to help to improve patients' knowledge level, especially among elders, through focusing on identifying risk factors to hypertension, regular drug intake, good nutrition, physical activity, and changing and informing lifestyles of patients with hypertension.

## Figures and Tables

**Table 1:** Percentage (%) of treatment and control of hypertension among hypertensive adults (*N* = 1836).

Variables	*N*	Control			Treatment		
		*n*	%	*P*	*n*	%	*P*
Gender							
Total	1863	572	31.2	—	1373	74.8	—
Male	722	222	30.7	*P* = 0.796	532	73.7	*P* = 0.383
Female	1114	350	31.4		841	75.5	
Age groups							
39–30	95	33	34.7	*P* = 0.135	56	58.9	*P* = 0.000
49–40	262	84	32.1		180	68.7	
59–50	534	183	34.3		401	75.1	
≥60	945	272	28.8		736	77.9	
Region							
Urban	822	308	37.5	*P* = 0.000	593	72.1	*P* = 0.019
Rural	1014	264	26		780	76.9	
Marital status							
Single	25	10	40	*P* = 0.251	16	64	*P* = 0.138
Married	1488	472	31.7		1104	74.2	
Divorced and widowed/widower	323	90	27.9		253	78.3	
Education level							
Illiterate	1149	322	28	*P* = 0.000	891	77.5	*P* = 0.004
Primary school	362	129	35.6		258	71.3	
Secondary school	114	34	29.8		76	66.7	
High school	46	22	47.8		32	69.6	
Diploma	90	28	31.1		60	66.7	
University	75	37	49.3		56	74.6	
Job							
Farmer	205	58	28.9	*P* = 0.351	143	71.1	*P* = 0.659
Employed	111	42	37.8		84	75.7	
Retired	184	64	34.8		137	74.5	
Unemployed	269	79	29.4		197	73.2	
Housekeeper	1071	329	30.7		812	75.8	
Income							
Poor	1655	495	29.9	*P* = 0.002	1235	74.6	*P* = 0.833
Medium	148	61	41.2		112	75.7	
Good	33	16	48.5		26	78.8	
Family history of hypertension							
Yes	832	289	34.7	*P* = 0.003	624	75	*P* = 0.845
No	1004	283	28.2		749	74.6	
Physical activity							
Yes	512	174	34	*P* = 0.104	380	74.2	*P* = 0.730
No	1324	398	30.1		993	75	
Tobacco							
Yes	253	61	24.1	*P* = 0.009	188	73.3	*P* = 0.852
No	1583	511	32.3		1185	74.9	
Body mass index (BMI)							
≤18.5	15	5	33.3	*P* = 0.612	11	73.3	*P* = 0.980
25–18.5	655	214	32.7		493	75.3	
30–25	736	229	31.1		547	74.3	
≥30	430	124	28.8		322	74.9	
Duration of diagnosis							
≤5	955	297	31.1	*P* = 0.045	669	70.1	*P* = 0.000
5–9	366	130	35.5		293	80.1	
10–14	298	79	26.5		235	78.9	
15–19	129	33	25.6		97	75.2	
≥20	88	33	37.5		79	89.8	

**Table 2:** Percentage (%) of knowledge of hypertension among hypertensive adults (*N* = 1836).

Variables	Weak		Average		Good		*P*
	*N*	%	*N*	%	*N*	%	
Gender							
Male	141	19.5	385	53.3	196	27.1	*P* = 0.261
Female	218	19.6	630	56.6	266	23.9	
Age groups							
39–30	22	23.4	39	41.1	34	35.8	*P* = 0.019
49–40	41	15.6	144	55	77	29.4	
59–50	100	18.7	300	56.2	134	25.1	
≥60	196	20.7	532	56.3	217	23	
Region							
Urban	149	18.1	452	55	221	26.9	*P* = 0.189
Rural	210	20.7	563	55.5	241	23.8	
Marital status							
Single	5	20	6	24	14	56	*P* = 0.004
Married	296	19.9	820	55.1	372	25	
Divorced and widowed/widower	58	18	189	58.5	76	23.5	
Education level							
Illiterate	234	20.4	663	57.7	252	21.9	*P* = 0.000
Primary school	72	19.9	206	57.9	84	23.2	
Secondary school	24	21.4	61	53.5	29	25.4	
High school	4	8.7	22	47.8	20	43.5	
Diploma	18	20	42	47.7	30	33.3	
University	7	9.3	21	28	47	62.6	
Occupation							
Farmer	35	17.4	114	56.7	52	25.9	*P* = 0.001
Employed	13	11.7	55	49.5	43	38.7	
Retired	34	18.5	88	47.8	62	33.7	
Unemployed	63	23.4	151	56.1	55	20.4	
Housekeeper	214	20	607	56.7	250	13.3	
Income							
Weak	322	20.1	20	13.5	7	21.7	*P* = 0.005
Average	927	56	73	49.3	15	45.5	
Good	396	23.9	55	32.2	11	33.3	
Family history of hypertension							
Yes	154	19.1	460	55.3	213	25.6	*P* = 0.874
No	200	19.9	555	55.3	246	24.4	
Tobacco							
Yes	33	27.5	66	55	21	17.5	*P* = 0.028
No	326	19	949	55.3	441	25.7	
Physical activity							
Yes	68	13.3	295	57.6	149	29.1	*P* = 0.000
No	291	22	720	54.4	313	23.6	
Body mass index (BMI)							
≤18.5	4	26.7	7	46.7	4	26.7	*P* = 0.157
25–18.5	143	21.8	364	55.6	148	22.6	
30–25	136	18.5	417	56.7	183	24.9	
≥30	76	17.7	227	52.7	127	29.5	
Duration of diagnosis							
≤5	183	19.2	522	54.7	250	26.2	*P* = 0.714
5–9	77	21	204	55.7	85	23.2	
10–14	60	20.1	169	56.7	69	23.2	
15–19	22	17.1	77	59.7	30	23.3	
≥20	17	19.3	43	48.9	28	31.8	
